# Rapid Annotation Strategy for *in Vivo* Phase II Metabolites of Anabolic–Androgenic Steroids Using Liquid Chromatography–Ion Mobility–Mass Spectrometry

**DOI:** 10.1021/jasms.5c00129

**Published:** 2025-07-23

**Authors:** David C. Koomen, Katrina L. Leaptrot, Jody C. May, Bailey S. Rose, Kyle E. Lira, Julia A. Raziel, Andrew D. Pumford, Gustavo de A. Cavalcanti, Monica C. Padilha, Henrique M. G. Pereira, John A. McLean

**Affiliations:** 1 Department of Chemistry, Center for Innovative Technology, 5718 Vanderbilt University, Nashville, Tennessee 37235, United States; 2 Brazilian Doping Control Laboratory (LBCD), 28125 Federal University of Rio de Janeiro (UFRJ), Rio de Janeiro, RJ 21941, Brazil

**Keywords:** untargeted metabolomics, LC-IM-MS, phase II AAS, nontargeted discovery workflow, CCS regression model, mass−mobility correlation

## Abstract

Doping control laboratories are responsible for the precise measurement of anabolic–androgenic steroids (AASs) and determination of athlete usage. Intact phase II AASs are difficult to analyze due to their low abundance in complex biological matrices and their structural similarities that convolute tandem mass spectrometry interpretation. Discovery efforts of unknown phase II metabolites of new-to-the-field steroids have been challenging due to these deficiencies in current analytical techniques. Several methods for determining unknown conjugated AAS compounds have been developed that include deuterium tagging, fractionation, derivatization, and utilization of synthesized standards. Ion mobility (IM), a rapid gas-phase separation, allows for improved molecular differentiation and provides additional information for analyzing intact phase II AASs without sacrificing throughput. Here, candidate metabolites were putatively identified for oxymetholone (OXM) and methyl-1-testosterone (M1T) utilizing liquid chromatography–ion mobility–mass spectrometry (LC-IM-MS) and two independent data analysis strategies: a fully untargeted approach using mass defect analysis and collision cross section (CCS) filtering and a pseudotargeted approach using the biologically anticipated isotopic envelope in conjunction with CCS filtering, temporal profiling, and tandem mass spectrometry confirmation. A proof-of-concept time-course study was conducted using the urine from healthy male individuals after steroid administration. The fully untargeted approach reduced the number of original features by >85% while the pseudotargeted approach reduced original features by >99%, yielding 11 possible novel phase II AAS candidates for OXM and 23 for M1T.

## Graphical abstract

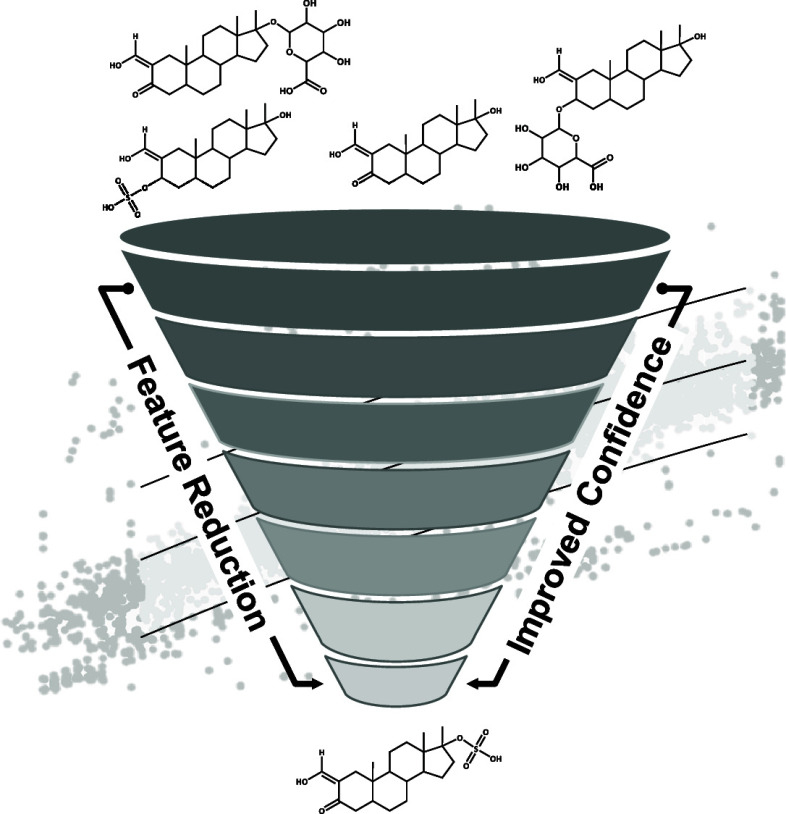

## Introduction

Anabolic–androgenic steroids (AASs) are synthesized derivatives of testosterone that promote skeletal muscle growth and have been misused as performance enhancing drugs by some athletes to gain an unfair advantage in competitive sports. After initial administration, exogenous AASs undergo phase I reactions, including oxidation, reduction, and hydroxylation, that alter the molecule for subsequent phase II reactions.^1,2^ Phase II AASs are long-term metabolites that can typically persist in urine for several weeks and are eliminated as conjugates incorporating polar functional groups such as sulfate or glucuronide.^3^ The addition of these polar moieties to AASs by enzymatically controlled conjugation reactions increases their water solubility and facilitates urinary excretion, providing optimal targets for tracking the misuse of AASs due to their ease of collection by a noninvasive method and their long-term stability *in vivo*.^1^ With over 95% of AASs measured by their phase II metabolite, premetabolized, or “free”, exogenous steroid molecules are rarely detected in urine samples.^4–6^ The minimum required performance level (MRPL) for analytical analysis is established by the World Anti-Doping Agency (WADA) for each exogenous AAS metabolite. This parameter aims to standardize the minimum required analytical performance of all accredited laboratories worldwide. Detection and identification of these compounds are essential for ensuring fair and equitable competition in athletics and must be closely monitored by doping control laboratories.

Mass spectrometry (MS) is a highly sensitive and selective analytical technique that can measure many compounds simultaneously. However, intact phase II AASs are difficult to analyze by MS due to their poor ionization efficiency, low abundance in biological samples, and interfering ion suppression arising from high chemical noise inherent to complex urine matrices.^7–9^ Traditionally, phase II AASs are analyzed by gas chromatography (GC)– or liquid chromatography–tandem mass spectrometry (LC-MS/MS); however derivatization reactions are often needed, and chromatographic runs limit throughput.^10–12^

Ion mobility spectrometry (IMS) can be coupled in line with LC and MS (LC-IM-MS) to provide additional selectivity that can potentially reduce the time scale of the chromatography stage, benefiting sample throughput. IMS is a gas-phase separation that differentiates ionized molecules based upon their shape, size, and charge and offers a strong advantage to improving analysis time comparatively to traditional GC- or LC-MS techniques used in routine doping control workflows.^9,13–16^ Previously, IMS techniques such as drift tube (DTIMS),^17^ traveling wave (TWIMS),^18^ and field asymmetric waveform ion mobility (FAIMS),^19^ have been used to analyze phase II AAS metabolites. In addition to faster, and in some cases, more selective separations compared to GC or LC, IMS provides a highly reproducible molecular descriptor in the form of ion-neutral collision cross section (CCS, Ω) values that are traceable to first principles.^15,20–23^ In addition to supporting compound identifications, CCS values can also be used to predict the putative identity of unknown compounds from empirical data of a similar class of molecules based upon regression models in conformational space from mass-mobility correlation analyses (*m*/*z* vs CCS).^24–26^ Thus, IM-MS measurements have been used to support spectral annotations through both direct database matching via mass and CCS, and chemical class prediction via correlation to mass-mobility regions of known compounds.

More recently, some athletes have resorted to different methods of AAS misuse to elude detection by using alternative (i.e., designer) steroids for sports doping that have not been established for testing by WADA.^27^ Misuse of these derivative compounds have become more prevalent, making discovery of novel phase II AASs difficult to establish for routine analysis.^28–31^ Additionally, there is an unmet need to develop analytical methods for discovering unknown phase II metabolites that are challenging to identify with GC- or LC-MS in their underivatized forms and with MS/MS where structurally similar precursors generate similar fragmentation spectra that are minimally informative. The detection and differentiation of intact phase II AAS isomers have been reported both by Davis et al. using LC-DTIMS-MS and by Rister et al. using TWIMS-MS metal adduction on free steroids; however, resolution of isomers of phase II AASs has been difficult to achieve for routine drug testing laboratories.^17,32^

Oxymetholone (OXM) and methyl-1-testosterone (M1T) are two steroids that have become more widely used, and their phase II metabolites have not been completely elucidated.^33–37^ The general structure for phase II AASs consists of 17 carbons arranged into a four-ring sterol backbone with sulfate and glucuronide conjugation sites at the 3 and 17 positions (Figure 1A).^1^ These two sites typically have hydroxyl groups that are stereoselective for phase II reactions with either an α or a β configuration. Glucuronide conjugation occurs at either a 3α or 17β hydroxyl while sulfate conjugation occurs at a 3β and some 17β hydroxylated AASs.^1^ Both OXM and M1T have 17β-hydroxyl groups that could undergo either a sulfate or glucuronide conjugation (Figure 1B,C). However, the 3-carbonyl on both of these compounds could also undergo phase I reactions that reduce it to either a 3α or 3β, allowing other possible sulfate or glucuronide conjugations to form for each compound. Therefore, the type of conjugation is difficult to quickly elucidate from the intact AAS structure alone, and previously described methods incorporate time-consuming techniques such as benchtop derivatization or online fractionation.^35,38,39^

**Figure 1:**
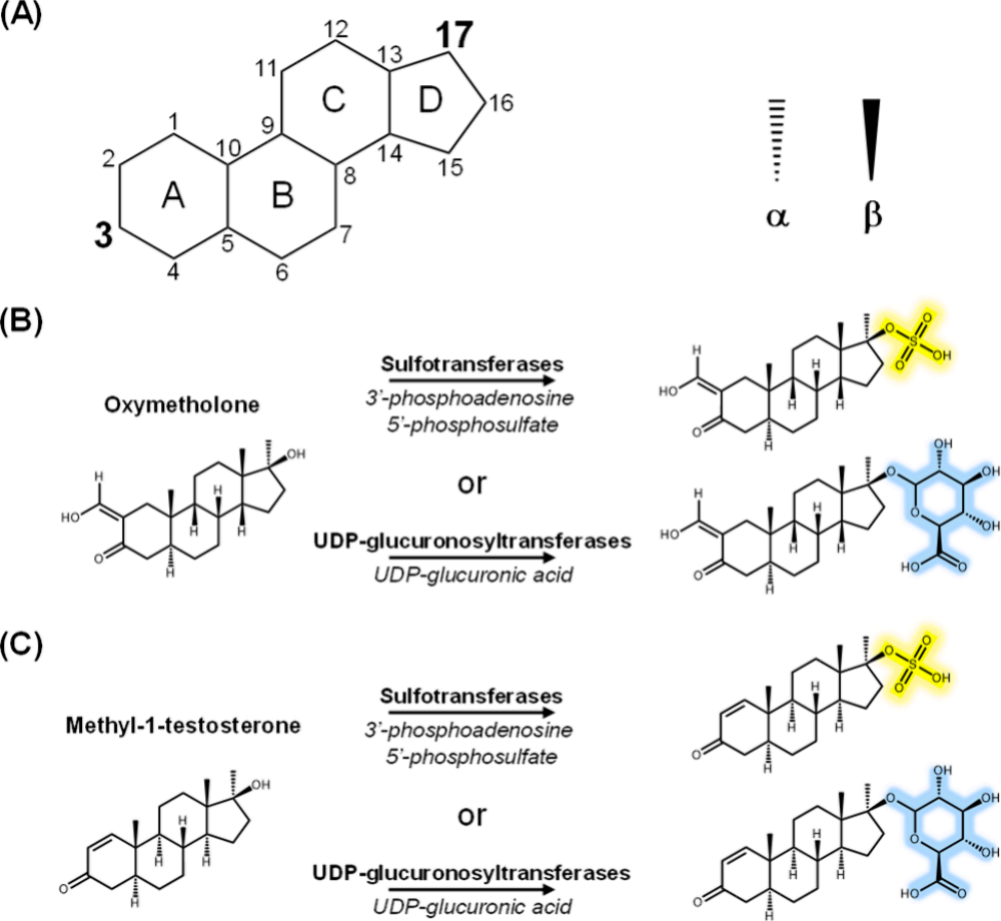
(A) Annotated sterol backbone structure for anabolic–androgenic steroids (AAS) with the possible conjugated sites numbered in bold and the nomenclature for AAS conjugate stereochemistry. (B) Structures for oxymetholone and (C) methyl-1-testosterone and examples of potential phase II sulfate (yellow) and glucuronide conjugates (blue) for each. Liver enzymes responsible for phase II bioconversion of exogenous compounds are denoted in bold above the arrow for the respective biotransformation (middle), with the respective substrates listed in italics below.

Several methods have been developed to discover the metabolites of new-to-the-field AASs. Detection of downstream, *in vivo* metabolites of 5α-androst-2-en-17-one was performed by Piper et al. using GC-thermal conversion-isotope ratio MS with heavy labeled deuterium tags, LC fractionation, and acetyl and trimethylsilyl (TMS) derivatization.^39^ Furthermore, unreported phase I metabolites of OXM have been characterized using TMS derivatization, LC fractionation, and GC-MS/MS analysis.^35^ For intact analysis of unknown phase II AASs, another analytical technique was described by Göschl et al. using an online solid-phase extraction-LC-high resolution MS/MS system with known synthesized standards of stanozolol-N-glucuronide.^38^ For compounds for which well-described phase II-conjugated standards are unavailable, such as OXM and M1T, these analytical discovery methodologies are insufficient for tentative identifications of downstream phase II metabolites. There are several possible metabolites and intermediates for these two steroids-of-interest. The metabolites of OXM have been previously studied, resulting in many possible reduction and oxidation products.^40^ Recently, Zheng et al. found a possible glucuronide metabolite for OXM, 2-methylene-17α-methyl-androstane-16ξ,17β-diol-3-one, using GC-Orbitrap-HRMS.^41^

Here, we use a fully untargeted 15 minute LC-IM-MS method without derivatization or fractionation as an alternative discovery methodology with the purpose of putatively identifying intact phase II metabolites of OXM and M1T. A postacquisition data filtering process was developed, in part, inspired by a filtering workflow for LC-IM-MS multiplexed untargeted metabolomics described by Reisdorph et al.^42^ To accomplish this, we use DTIMS coupled to LC and MS for the multidimensional separation (LC-IM-MS) of AASs. Furthermore, to investigate the elimination of OXM and M1T over time, independent time-course experiments were conducted by collecting urine samples from healthy adult males after administration. To investigate possible secondary metabolites of these steroids, two discovery data analysis workflows were developed to identify potential AAS conjugates: (i) a fully untargeted approach and (ii) a pseudotargeted data analysis approach. The fully untargeted approach uses parameters derived from the primary analytical measurements (e.g., mass–mobility correlation and mass defect) of chemical standards from the same structural class to enable tentative identification of intact phase II AASs. For the pseudotargeted approach, temporal metabolic profile samples are considered for further filtering with subsequent LC-IM-MS/MS analysis to confirm the putative identification of an AAS metabolite by the presence of phase II-indicative moieties.

## Experimental Methods

### Standards and Chemicals

Standards of 20 phase II sulfate and glucuronide anabolic androgenic steroids were obtained from Steraloids (Newport, RI) and The National Measurement Institute of Australia (NMIA). Stanozolol 1′N-G was provided by Seibersdorf Laboratories (Austria), and Epi-THMT S3 was a gift from the Institute Hospital del Mar d’Investigacions Mèdiques (IMIM) (Barcelona, Spain). Optima LC-MS grade water, methanol, formic acid, and ammonium formate were purchased from Fisher Scientific (Hampton, NH).

### Drug Administration and Preparation of Human Urine Samples

Oxymetholone (OXM) and methyl-1-testosterone (M1T) were administered separately to one healthy adult male at 150 mg and 10 mg, respectively, and urine samples were collected over a period of 84 h. Standards in blank urine and metabolites of OXM and M1T in test-subject urine were isolated by solid-phase extraction (SPE-C18 Cartridges) with an initial conditioning of 2 mL of methanol and 2 mL of water and then loaded with 5 mL of human urine. Eluates were then dried under nitrogen gas for 40 min at 40 °C and reconstituted in 100 μL of 45% aqueous methanol.

### Chromatographic and DTIMS-MS Conditions

Blank urine, spiked blank urine, and urine collected after OXM and M1T administration were analyzed using multidimensional LC-IM-MS incorporating a 1290 Infinity LC system coupled to a 6560 drift tube ion mobility Q-TOF (Agilent Technologies, Santa Clara, CA). Steroid standards spiked in blank urine and human urine samples containing OXM and M1T were separated on a 2.1 mm × 75 mm Waters ACQUITY BEH C_18_ reversed-phase column with a 2.1 mm × 5 mm Waters ACQUITY BEH C_18_ Vanguard precolumn, both with a 1.7 μm particle size. Mobile phases A and B were water and methanol, respectively, with 0.1% formic acid and 1 mM ammonium formate additives. The chromatographic run consisted of an injection volume of 10 μL at a flow rate of 400 μL/min maintained at 45 °C. The 15 min LC gradient began with 45% B for 1 min, ramping to 70% B over 8.5 min and then increased to 100% B for 1 min, held for 1.5 min, and then decreased to 45% B for 1 min and held for 2 min (equilibration time).

Samples and AAS standards were analyzed in the negative ionization mode. Preliminary analyses of AAS standards provided ionization for glucuronide AASs in both positive and negative ionization mode, while sulfate AAS standards ionized solely in negative mode. Therefore, to simultaneously capture unknown candidate features for both of these structural moieties, the data acquisition was conducted in negative mode. The following electrospray ionization source conditions were used: gas temp, 300 °C; drying gas, 12 L/min; nebulizer pressure, 20 psi; sheath gas temperature, 300 °C; sheath gas flow, 12 L/min; capillary voltage, 2000 V; nozzle voltage, 500 V; MS TOF fragmentor, 340 V; octapole RF *V*_pp_, 750 V. DTIMS analysis was performed in single pulse mode with nitrogen gas at a temperature of ∼30 °C, a pressure of 3.94 Torr, and a drift field of 16.0 V/cm. The drift tube was operated in single pulse mode, although we note that multiplexed mode would afford higher sensitivity while enabling access to high resolution demultiplexing (HRdm). Additional DTIMS-MS parameters were as follows: mass range, *m*/*z* 50–1700; trap fill time, 40 000 μs; trap release time, 200 μs; frame rate, 0.9 frames/s; IM transient rate, 18 IM transients/frame; max drift time, 60 ms; TOF transient rate, 600 transients/IM transients; drift tube entrance, −1472 V; drift tube exit, −222 V; rear funnel entrance, −215.5 V; rear funnel exit, −43 V. Reference A (purine and hexakis­(2,2,3,3-tetrafluoropropoxy)­phosphazene) of the calibrant delivery system was continuously infused into the secondary nebulizer during LC-IM-MS analyses for accurate mass calibration postacquisition. For LC-MS analysis, the IM stage was operated in a QTOF-only (Rp capable of ∼40 000) “pass through” mode by disengaging the ion trap-and-release functionality, with all other settings remaining the same.

### MS/MS Analysis

The same electrospray conditions and instrument parameters outlined above were used for MS/MS analysis, with the targeted MS/MS inclusion list having the following parameters: MS transients/spectrum, 8127; MS/MS transients/spectrum, 8043; delta retention time, 0.2 min; isolation width, narrow *m*/*z* ∼1.3; collision energy, 30 V. Standards spiked in blank urine were prepared at 5 μg/mL for MS/MS analysis. Time collection points were pooled for the MS/MS analysis of unknown metabolites. Two separate runs were used with separate inclusion lists, where more than one potential candidate feature fell within the same retention time window.

### Data Analysis and Software

Data files were first processed with MassHunter IMS Reprocessor (Agilent) and PNNL PreProcessor.^43^ Features were extracted from the processed datafiles using the IMFE algorithm implemented in Mass Profiler 10.0 (Agilent) which generates a list of molecular features, which are deisotoped triplets of discrete RT-CCS-*m*/*z*. Putative identifications were matched to theoretically expected isotopic distributions with PCDL Manager (Agilent). Collision cross section (CCS) measurements were obtained using the single field calibration described by Stow et al.^22^ CCS values for mass–mobility correlation were curated using the Unified CCS Compendium.^25^ The signal intensity for each feature was normalized to the total ion chromatogram of each respective sample. The signal intensity for Figure 2 was normalized to 19-norandrosterone-D4 glucuronide, which was spiked in with the standards in blank urine in a separate experiment from the OXM and M1T time course.

**Figure 2:**
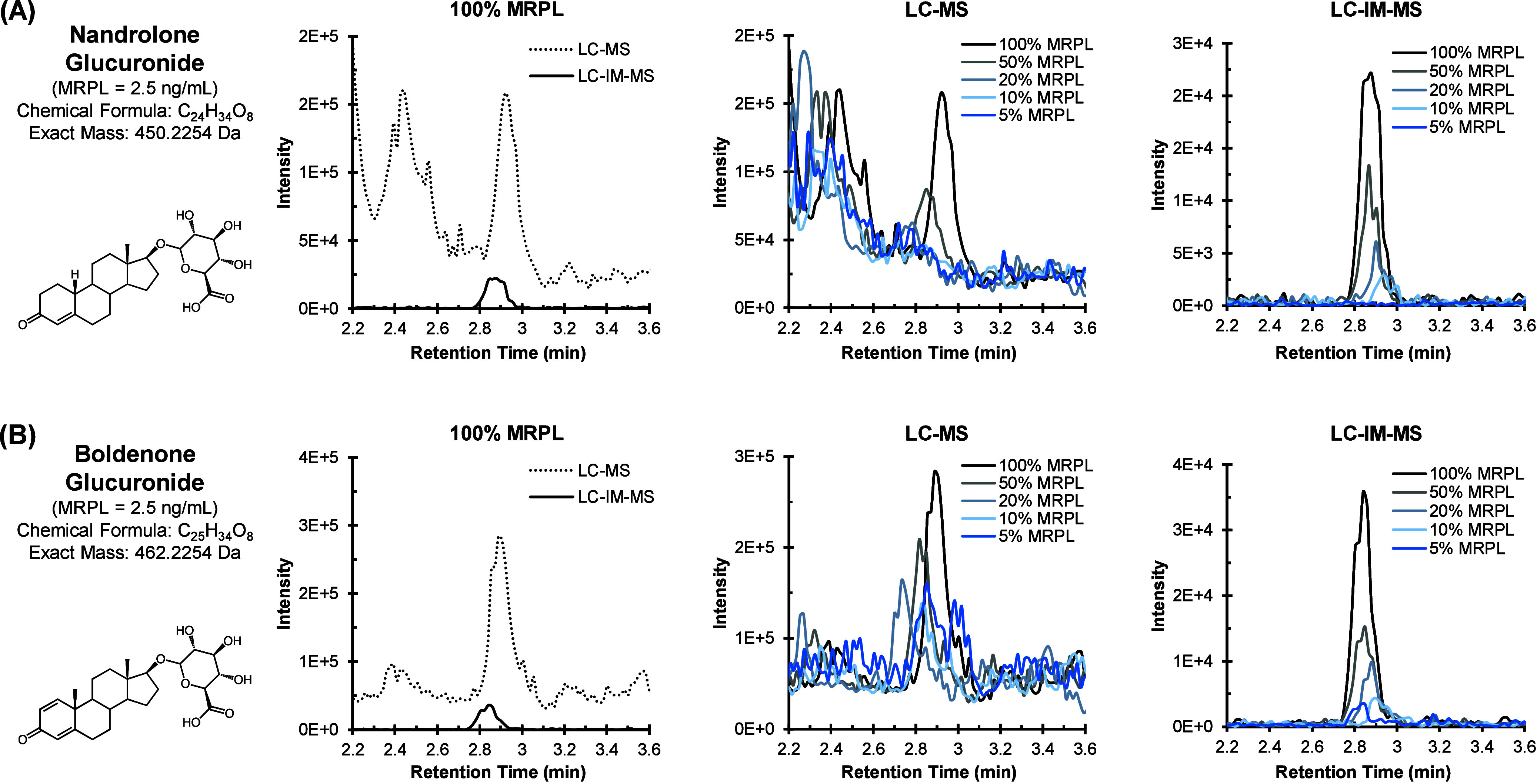
(A) Extracted ion chromatograms for nandrolone glucuronide and (B) boldenone glucuronide, comparing LC-MS and LC-IM-MS analyses of standards spiked in blank urine. Concentrations of each analyte were measured at 5%, 10%, 20%, 50%, and 100% of the MRPL for each analyte. Chromatograms are normalized to 19-norandrosterone-D4 glucuronide.

## Results and Discussion

Sulfate and glucuronide AAS standards spiked in blank urine were first analyzed to assess the multidimensional selectivity of the LC-IM-MS system for the phase II AASs. The extracted ion chromatograms of two exemplary cases, boldenone glucuronide and nandrolone glucuronide, are presented in Figure 2. Here, both AAS standards exhibit reduced chemical noise and improved selectivity, with a corresponding reduction in total signal by approximately 1 order of magnitude when operating LC-IM-MS as compared with LC-MS on the same platform. This loss in sensitivity is typical for IM and is an expected consequence of operating an additional ion gate in line with the ion beam, though much of this signal could be recovered if the IM stage were operated in a multiplexed mode.^44–46^ Concentrations for each compound were measured at 100%, 50%, 20%, 10%, and 5% of the initial minimum required performance level (MRPL, 2.5 ng/mL). For conventional LC-MS analysis, significant ion suppression and matrix effects are observed for each compound at varying MRPL concentrations, corresponding to low signal-to-noise ratios and a shift in the retention time (RT) as the analyte concentration is reduced. However, for LC-IM-MS analysis, the signal-to-noise ratio remains high for all but the lowest concentration (5% MRPL) evaluated, and the extracted ion chromatograms exhibit minimal shift in RT across the full concentration range surveyed. Irrespective of the observed loss in sensitivity, the added selectivity of IM was found to improve the analysis of AASs in urine matrices by enhancing the signal-to-noise ratio, limits of detection, and RT reproducibility.

Glucuronide conjugates are structurally larger than sulfate conjugates and therefore have measurably larger CCS values with IM analysis.^17^ This inherent difference in molecular size can be used to predict whether an unknown feature is likely to be functionalized with either a sulfate or a glucuronide moiety. To this end, two approaches were developed to identify possible phase II metabolites resulting from AAS exposure: a fully untargeted discovery approach and a pseudotargeted prioritization approach (Figure 3A). The fully untargeted approach aims to identify unknown metabolites containing similar conjugate molecules by initially applying an IM filter trained from known steroid CCS values hosted in the Unified CCS Compendium and *m/*z values of steroids and steroid conjugates in the LIPID MAPS Structural Database (LMSD).^25,26,47^ For this filtering step, CCS regression models from empirical data of a similar subclass of compounds were developed to aid in predicting the identification of unknown compounds (Figure 3B and Figure S1A). Additionally, a mass defect analysis using the Kendrick scale for known steroid molecular formulas was applied as an additional filter for rejecting features outside the 99% predictive interval and *m*/*z* of the model (Figure 3B and Figure S1B).^48,49^

**Figure 3:**
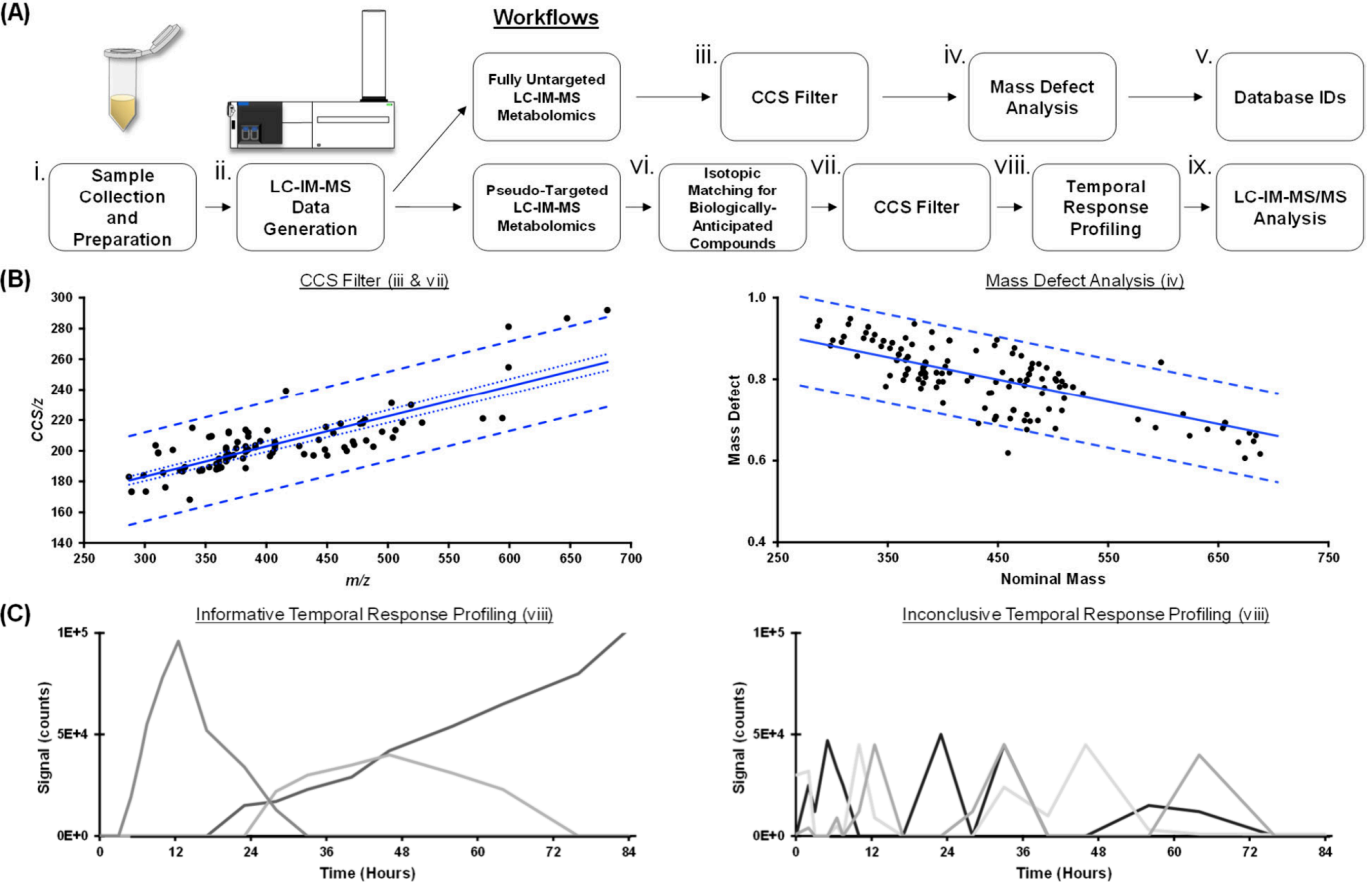
(A) Flow diagram depicting the experimental outline and data interrogation strategies. (B) Linear regression models for ion mobility conformational space and mass defect derived from empirical measurements of steroids and steroid conjugates (black dots) from the Unified CCS Compendium with the predictive interval (large dash), confidence interval (small dash), and mean (solid line). (C) Examples of temporal response profiling trends of candidate features illustrating informative pharmacokinetic changes of compounds over time (left) vs less conclusive response curves of unlikely candidates (right).

The pseudotargeted approach utilized a list of chemical formulas of biologically anticipated sulfate and glucuronide conjugates derived from knowledge of the parent compounds (Table S1). Features present in the blank urine were first rejected via blank subtraction, and remaining features were tentatively identified based upon matching *m*/*z* and the theoretical isotopic envelopes. Then, using the empirically known mass–mobility correlation of the steroid entries within the Unified CCS Compendium, feature CCS values that were within the predictive interval (where 99% of theoretical values for steroids would occur) were prioritized as likely to be phase II conjugates of the precursor AAS (Figure 3B).^23,25^ Furthermore, the time-resolved profiles of these putatively identified compounds were used to improve the confidence. Example temporal profiles in Figure 3C exhibit an informative response, indicated by a gradual increase of signal over time. However, contradictory to the informative temporal profiles, inconclusive response profiles with inconsistent signals over time suggest an unlikely candidate feature. Collectively, these strategies were applied to two AAS study cases, oxymetholone and methyl-1-testosterone, to demonstrate the filtering capabilities of these approaches.

The number of features reduced by both strategies is summarized in Figure 4 for oxymetholone (OXM) and methyl-1-testosterone (M1T). Each data set for OXM and M1T began with an initial number of features (*n* > 10 000). For the fully untargeted filtering approach, the number of features is reduced after blank subtraction to 7369 and 6397 for OXM and M1T, respectively. Features for both case studies were then reduced by an additional 15% using CCS values within the predictive interval and subsequently reduced by an additional 3% with an iterative mass defect filter. To further prioritize candidate features, a frequency cutoff of ≥3 was used, which reduced the number of features by 23% for OXM and 43% and M1T. The term frequency here refers to the number of samples across the time course that exhibited a signal greater than 0. The remaining features were then identified using CCS values of AASs hosted in the Unified CCS Compendium and *m*/*z* values of steroids and steroid conjugates in LMSD.^23,25,47^ This resulted in 1659 and 1014 identified features for OXM and M1T, respectively. While the fully untargeted approach (Figure 4A) narrowed the possible identifications by 85% or more for both AAS examples, the number of prioritized features was still too large (*n* > 1000) to create inclusion lists for MS/MS confirmation analysis. This observation initially motivated the development of the pseudotargeted approach, with the objective of providing a more rigorous, yet specific, reduction of features to candidate numbers suitable for tandem mass spectrometry (MS/MS).

**Figure 4:**
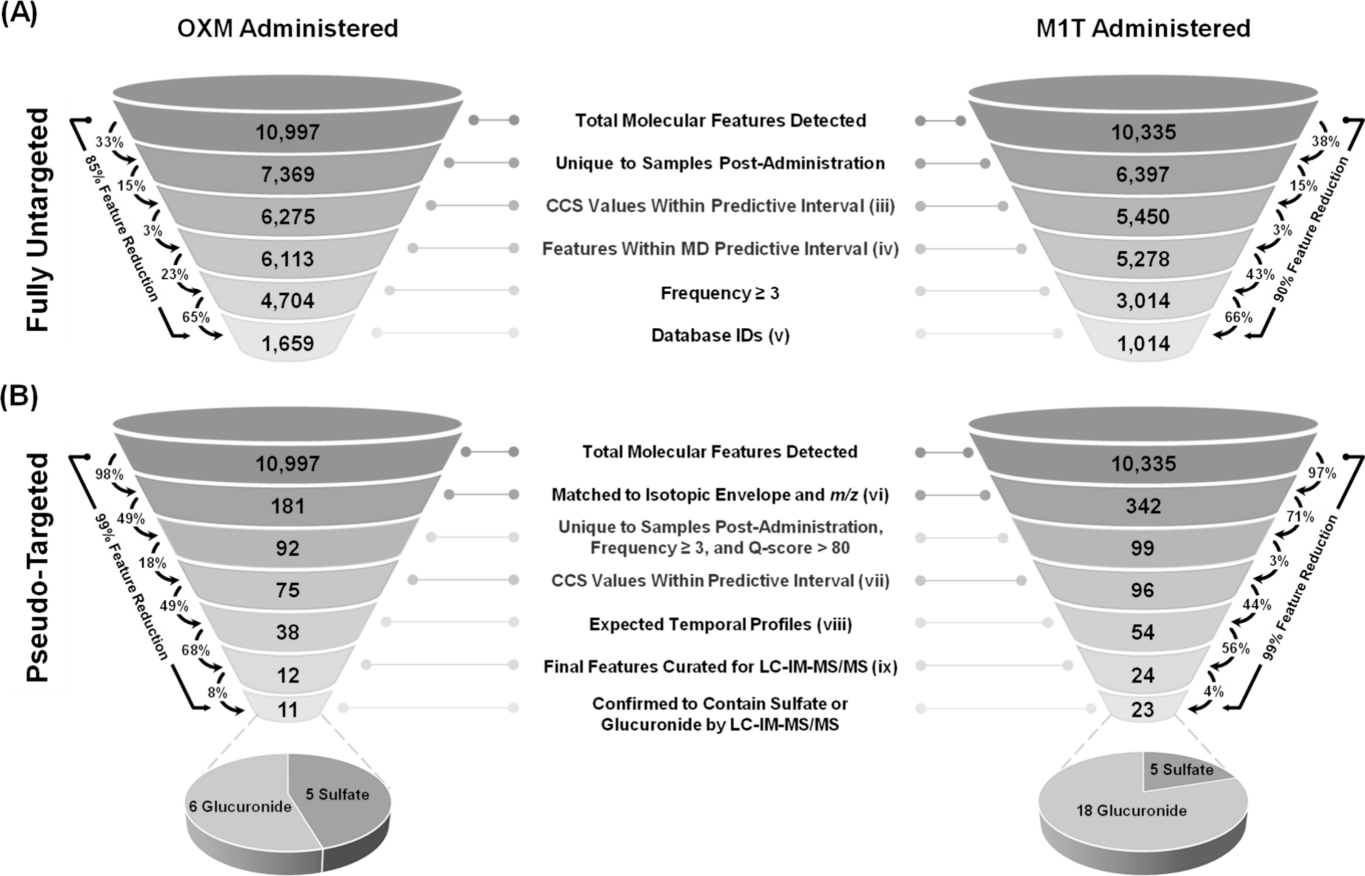
Iterative steps taken for reducing the initial pool of detected molecular features from oxymetholone (OXM) and methyl-1-testosterone (M1T) administration using both the (A) fully untargeted and (B) pseudotargeted filtering methods.

Independent of the fully untargeted strategy, the pseudotargeted filtering approach reduced the features by ≥97% for both data sets (Figure 4B) by searching for the isotopic envelope of biologically anticipated sulfate and glucuronide conjugates of the precursor AAS (Table S1).^34,35^ This resulted in 181 and 342 features for OXM and M1T that could be more closely investigated as possible phase II metabolites of the precursor AAS. Features unique to the sample postadministration, with a frequency of three or greater across the time points and with a *Q*-score of >80, yielded 92 and 99 features for OXM and M1T, respectively. Using a 99% predictive interval for aligning the measured CCS to the mass–mobility correlation band (*m*/*z* vs CCS) from the phase II AAS standards in the Unified CCS Compendium reduced the number of features to 75 for OXM and 96 for M1T. This IM filtering step was more useful for the OXM data set than the M1T with an 18% vs 3% reduction of features. The number of features with an informative temporal response profile yielded a further reduction of features to 38 for OXM and 54 for M1T. Finally, 12 candidate features for OXM and 24 candidate features for M1T were manually curated using their temporal response profiles to create an inclusion list for MS/MS.

Here, an initial analysis of the time-course data (Figure 5) revealed that candidate OXM sulfate [M – H]^−^ and OXM M1 glucuronide [M – H]^−^ metabolites demonstrated temporal profiles characterized by a gradual increase of signal over time, correlating to a metabolic signature associated with the bioconversion of an original or phase I compound into the phase II candidate feature. In the M1T time-course, THMT-13 sulfate [M – H]^−^ and THMT glucuronide [M – H]^−^ also exhibited promising diagnostic temporal profiles (Figure S3). Using these corresponding pieces of analytical information (mass accuracy, isotopic envelope alignment, CCS correlation, and diagnostic temporal profiles), a final set of features for both the OXM and M1T compounds was curated for tandem mass spectrometry MS/MS analysis.

**Figure 5:**
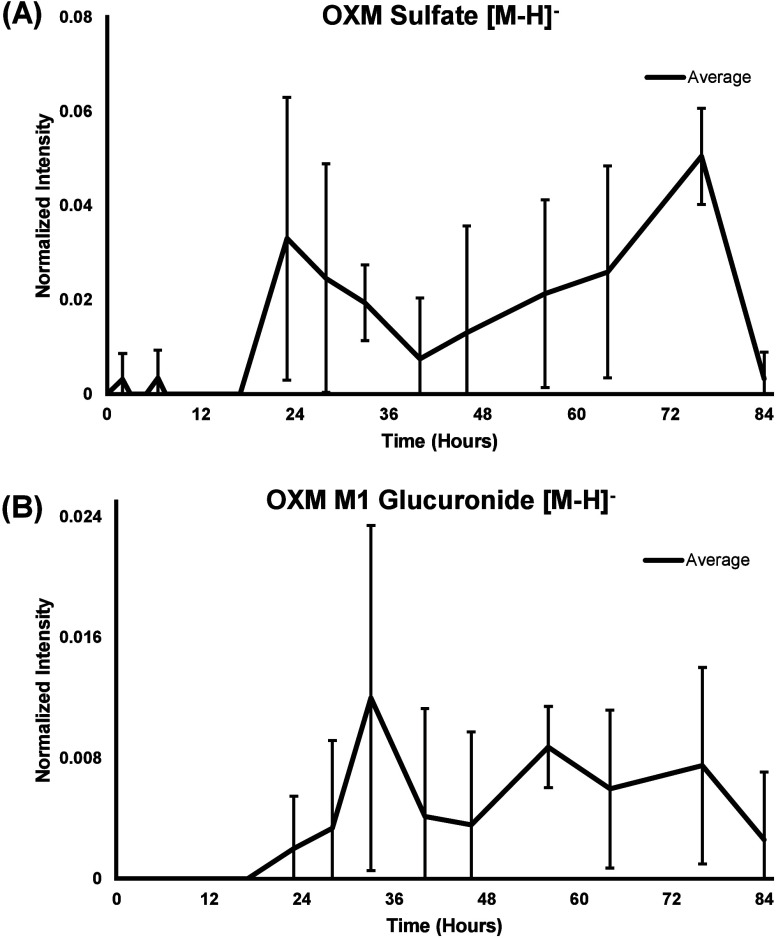
Temporal response profiles for example (A) sulfate and (B) glucuronide metabolites of oxymetholone (OXM). Standard deviations were calculated from technical replicates (three instrument injections). Intensity values are normalized to the total ion chromatogram. Low intensities resulted in large standard errors.

While MS/MS experiments using collision-induced dissociation (CID) are insufficient to distinguish between structural isomers and stereoisomers of phase II AASs, CID is widely implemented and can inform broadly whether or not the molecular features contain a glucuronide or sulfate moiety (Figure S4). A total of 9 glucuronide and 13 sulfate phase II AAS standards were first analyzed by MS/MS to assess the method’s ability to determine the presence of a glucuronide or sulfate and to evaluate the collision energy needed for broad fragment ion coverage (Figure S2). Intact sulfate AAS standards demonstrated transitions from precursor ions to *m*/*z* 97 and *m*/*z* 79 fragments, diagnostic of a hydrogen sulfate and a sulfonic acid group, respectively. Intact glucuronide AAS standards exhibited three diagnostic fragments at *m*/*z* 113, *m*/*z* 85, and *m*/*z* 75, with some glucuronide precursor ions also exhibiting a neutral loss (NL) of 176 Da (Figure S4). The sulfate and glucuronide fragments observed for these standards were consistent with previous literature.^50–53^ From these results, a consensus collision energy of 30 V (laboratory frame) was found to provide broad-scale fragmentation coverage for both sulfate and glucuronide standards, and therefore, 30 V CID was subsequently used for comprehensive analysis of all unknowns in the OXM and M1T inclusion lists created from the pseudotargeted filtering approach.

Inclusion lists were populated with the RT and *m*/*z* from the filtered feature list for each of the putative metabolites identified as final candidates in the OXM and M1T data sets. Of the 12 putative identifications for the OXM, 11 (∼92%) were determined to contain either a sulfate or glucuronide (Figure 4B). For M1T, 23 out of 24 (∼96%) putative identifications contained either a sulfate or a glucuronide (Figure 4B). For OXM, 6 out of those 11 candidate identifications were glucuronide and 5 were sulfate, while the 23 putative identifications for M1T comprised 18 glucuronide-containing and 5 sulfate-containing metabolites (Table S2 and Table S3). Example MS/MS spectra for feature candidates can be found in Figure S4. In contrast to the fully untargeted approach that filtered out 90% of the features, the pseudotargeted approach filtered out 99.9% and 99.8% of features for OXM and M1T, respectively, leading to several potential candidates for possible conjugated products that were confirmed to contain either sulfate or glucuronide moieties based upon structural MS/MS analysis.

## Conclusions

The workflows demonstrated in this study provide an additional level of confidence for identifying phase II AASs where full validation is challenging due to the high number of possible structural analogs including stereoisomers and the corresponding lack of analytical standards. This work aims to establish workflows for quickly annotating possible candidate features for phase II metabolism of precursor molecules present in complex matrices such as urine. However, additional testing with well-characterized synthetic standards or stereochemical annotation with data from nuclear magnetic resonance would need to be performed to confirm these results. Further improvements to the fully untargeted workflow could be made by incorporating machine learning, such as SIFTER, which was specifically designed to implement compound class prediction integrating *m*/*z*, CCS, and mass defect, or by incorporating HRdm annotation workflows for potentially improved isomer separation of unknown features.^54^ In addition to HRdm, other high-resolution ion mobility platforms, including structures for lossless ion manipulation, trapped ion mobility, or cyclic ion mobility, could be utilized in conjunction with LC-MS to improve peak capacity. Future work could also include longer sample collection periods over several weeks to assess long-term metabolites and/or more biological samples for validating the unknown metabolites of each of these designer steroids in physiological systems. In regard to quantitative aspects utilizing IM for AAS analysis, the Chouinard group has demonstrated limits of detection in the sub ng/mL range for select AAS demonstrating feasibility for eventual IM-incorporated analyses of AASs in routine testing.^9,55^ Whereas further validation and examination would be required to ensure that these compounds could be used for routine testing by doping control laboratories, this work provides a promising analytical strategy for addressing the chemical complexity present in these steroid analyses.

## Supplementary Material



## Notes

Proposed candidate features, filtered metabolite candidates, untargeted data analysis results, structural information for AAS standards, and temporal graphs for M1T and MS/MS spectra (PDF)

## Abbreviations

AAS: anabolic androgenic steroid
CCS: collision cross section
CID: collision induced dissociation
DTIMS: drift tube ion mobility spectrometry
FAIMS: field asymmetric waveform ion mobility spectrometry
GC: gas chromatography
IMS: ion mobility spectrometry
LC: liquid chromatography
LC-IM-MS: liquid chromatography–ion mobility–mass spectrometry
*m*/*z*: mass-to-charge ratio
M1T: methyl-1-testosterone
MRPL: minimum required performance level
MS/MS: tandem mass spectrometry
OXM: oxymetholone
RT: retention time
SIFTER: supervised inference of feature taxonomy from ensemble randomization
TWIMS: traveling wave ion mobility spectrometry
WADA: World Anti-Doping Agency
