# Supplementary material for: Rapid Annotation Strategy for in Vivo Phase II Metabolites of Anabolic–Androgenic Steroids Using Liquid Chromatography–Ion Mobility–Mass Spectrometry
Source: J Am Soc Mass Spectrom. 2025 Jul 23;36(8):1762–70. doi: 10.1021/jasms.5c00129 (PMC12333374; doi:10.1021/jasms.5c00129)
Supplement: Supplementary file 1 [file js5c00129_si_001.pdf]

Supporting Information

Rapid Annotation Strategy for *in vivo* Phase II Metabolites of Anabolic-Androgenic Steroids using Liquid Chromatography-Ion Mobility-Mass Spectrometry

David C. Koomen,[†] Katrina L. Leaptrot,[†] Jody C. May,[†] Bailey S. Rose,[†] Kyle E. Lira,[†] Julia A. Raziel,[†] Andrew D. Pumford,[†] Gustavo de A. Cavalcanti,[‡] Monica C. Padilha,[‡] Henrique M. G. Pereira[‡] and John A. McLean[†*]

[†] Department of Chemistry, Center for Innovative Technology, Vanderbilt University, Nashville, Tennessee, USA 37235
[‡] Brazilian Doping Control Laboratory - LBCD - Federal University of Rio de Janeiro - UFRJ, Rio de Janeiro, RJ, Brazil 21941

*Email: john.a.mclean@vanderbilt.edu

Supplemental Figures:

Table S1. Proposed expected conjugates of oxymetholone and methyl-1-testosterone
Table S2. Final filtered candidate features for oxymetholone
Table S3. Final filtered candidate features for methyl-1-testosterone
Figure S1. Fully untargeted filtering plots for mass-mobility correlation and mass defect
Figure S2. Structures for 22 phase II AAS standards used for MS/MS optimization
Figure S3. Methyl-1-testosterone example temporal profiles
Figure S4. MS/MS spectra for example standards and example OXM and M1T compounds from inclusion list

Table S1. Biologically-anticipated conjugates proposed for oxymetholone and methyl-1-testosterone with their chemical formula, molecular weight, and deprotonated mass-to-charge ratio. IUPAC names for some of the following short-hand terms in the table are as follows: THMT S2, 3α-sulphate-5α-androstan-17β-methyl-17α-ol; THMT S3, 3α-sulphate-5β-androstan-17β-methyl-17α-ol; THMT, 17α-Methyl-5β/5α-androstane-3α,17β-diol; Epi-THMT, 17β-Methyl-5β/5α-androstane-3α,17α-diol; THMT-13, 18-Nor-17β-hydroxymethyl-17α-Methyl-5β/5α-androstane-13-en-3α-ol; Oxymetholone (OXM), 17β-hydroxy-2-hydroxymethylene-17α-methyl-5 alpha-androstan-3-one; OXM M1, 2ξ-hydroxymethyl-17α-methyl-5α-androstan-17β-ol, 3-one or 2-hydroxymethylene.

Biologically-Anticipated Compound	Formula	Molecular Weight	$[M-H]^-$
Oxymetholone	$C_{21}H_{32}O_3$	332.2351	331.2278
OXM M2 Glucuronide	$C_{27}H_{40}O_8$	492.2723	491.2650
OXM M2 Sulfate	$C_{21}H_{32}O_5S$	396.1970	395.1897
OXM Sulfate	$C_{21}H_{32}O_6S$	412.1920	411.1847
OXM Glucuronide	$C_{27}H_{40}O_9$	508.2672	507.2599
OXM bis-glucuronide	$C_{33}H_{48}O_{15}$	684.2993	683.2920
OXM bis-sulfate	$C_{21}H_{32}O_8S_2$	476.1539	475.1466
OXM bis-sulfate V2	$C_{21}H_{32}O_9S_2$	492.1488	491.1415
OXM M1 Glucuronide	$C_{27}H_{42}O_9$	510.2829	509.2756
OXM M1 bis-glucuronide	$C_{33}H_{50}O_{15}$	686.3150	685.3077
OXM M2 bis-sulfate	$C_{21}H_{34}O_8S_2$	478.1695	477.1622
Methyl-1-testosterone	$C_{20}H_{30}O_2$	302.2246	301.2173
Epi-THMT S2 or S3 (RM);	$C_{20}H_{34}O_5S$	386.2127	385.2054
Epi-THMT S2 or S3 (RM), water loss	$C_{20}H_{32}O_4S$	368.2021	367.1948
M1T Glucuronide	$C_{26}H_{38}O_8$	478.2567	477.2494
THMT Glucuronide	$C_{26}H_{42}O_8$	482.2880	481.2807
THMT bis-Glucuronide	$C_{32}H_{50}O_{14}$	658.3201	657.3128
THMT bis-Sulfate	$C_{20}H_{34}O_8S_2$	466.1695	465.1622
THMT-13 Sulfate	$C_{20}H_{32}O_5S$	384.1970	383.1897
M1T-13 Glucuronide	$C_{26}H_{36}O_8$	476.2410	475.2337
THMT-13 Glucuronide	$C_{26}H_{40}O_8$	480.2723	479.2650
M1T-13 Sulfate	$C_{20}H_{28}O_5S$	380.1657	379.1584

Table S2. Metabolite candidates from filtering criteria for oxymetholone. Features were exported from Agilent Mass Profiler.

Candidate Name	Formula	Ion Species	Molecular Weight	RT	SD	DT	SD	CCS	SD	m/z	SD	Abundance	Freq.	Q Score	MS/MS?
OXM Sulfate	$C_{21}H_{32}O_6S$	$(M\text{-}H)^-$	412.1920	10.497	0.046	27.883	0.122	198.91	0.57	411.1854	0.0011	2520	12	100	Y
OXM Sulfate	$C_{21}H_{32}O_6S$	$(M\text{-}H)^-$	412.1920	10.245	0.060	27.940	0.100	199.13	0.61	411.1856	0.0017	3477	20	100	Y
OXM Sulfate	$C_{21}H_{32}O_6S$	$(M\text{-}H)^-$	412.1920	2.908	0.116	29.169	0.088	207.83	0.50	411.1848	0.0008	4901	32	100	Y
OXM Sulfate	$C_{21}H_{32}O_6S$	$(M\text{-}H)^-$	412.1920	1.858	0.082	29.307	0.117	208.80	0.79	411.1857	0.0008	2520	35	100	Y
OXM M2 bis-sulfate	$C_{21}H_{34}O_8S_2$	$(M\text{-}H\text{-}H2O)^-$	478.1695	1.377	0.060	27.333	0.079	194.15	0.31	459.1511	0.0007	5167	30	97.3	Y
OXM Glucuronide	$C_{27}H_{40}O_9$	$(M\text{-}H)^-$	508.2672	6.384	0.159	30.092	0.096	213.43	0.51	507.2628	0.0013	493	8	82.4	Y
OXM Glucuronide	$C_{27}H_{40}O_9$	$(M\text{-}H)^-$	508.2672	10.92	0.041	30.523	0.030	216.54	0.21	507.2610	0.0005	1132	10	99.3	Y
OXM Glucuronide	$C_{27}H_{40}O_9$	$(M\text{-}H)^-$	508.2672	10.834	0.071	30.783	0.114	217.94	0.45	507.2606	0.0012	1710	25	90	Y
OXM Glucuronide	$C_{27}H_{40}O_9$	$(M\text{-}H)^-$	508.2672	10.723	0.047	30.909	0.023	218.54	0.19	507.2604	0.0008	1020	18	83.8	Y
OXM M1 Glucuronide	$C_{27}H_{42}O_9$	$(M\text{-}H)^-$	510.2829	4.554	0.045	31.585	0.088	223.35	0.74	509.2748	0.0009	850	14	97.9	Y
OXM M1 Glucuronide	$C_{27}H_{42}O_9$	$(M\text{-}H)^-$	510.2829	10.425	0.034	31.829	0.144	225.12	0.79	509.2757	0.0008	3838	21	97	Y
OXM M1 Glucuronide	$C_{27}H_{42}O_9$	$(M\text{-}H\text{-}H2O)^-$	510.2829	11.024	0.035	31.333	0.145	221.94	0.68	491.2668	0.0012	1369	15	100	N

Table S3. Metabolite candidates from filtering criteria for methyl-1-testosterone. Features were exported from Agilent Mass Profiler.

Candidate Name	Formula	Ion Species	Molecular Weight	RT	SD	DT	SD	CCS	SD	m/z	SD	Abundance	Freq.	Q Score	MS/MS?
M1T-13 Sulfate	$C_{20}H_{28}O_5S$	$(M-H)^-$	380.1657	0.902	0.041	25.746	0.101	184.02	0.71	379.1609	0.0010	1085	9	91.8	Y
THMT-13 Sulfate	$C_{20}H_{32}O_5S$	$(M-H)^-$	384.1970	6.612	0.066	28.752	0.033	204.64	0.28	383.1888	0.0009	999	7	100	Y
THMT-13 Sulfate	$C_{20}H_{32}O_5S$	$(M-H)^-$	384.1970	9.498	0.023	27.21	0.029	195.00	0.21	383.1906	0.0006	679	5	96.9	Y
Epi-THMT S2 or S3 (RM)	$C_{20}H_{34}O_5S$	$(M-H)^-$	386.2127	4.933	0.099	28.395	0.057	202.15	0.26	385.2046	0.0006	2240	14	100	Y
Epi-THMT S2 or S3 (RM)	$C_{20}H_{34}O_5S$	$(M-H)^-$	386.2127	5.204	0.097	28.293	0.078	202.22	0.13	385.2054	0.0010	1543	11	100	Y
M1T-13 Glucuronide	$C_{26}H_{36}O_8$	$(M-H-H2O)^-$	476.2410	6.297	0.091	31.619	0.053	223.69	0.36	457.2258	0.0009	3189	21	100	Y
M1T-13 Glucuronide	$C_{26}H_{36}O_8$	$(M-H-H2O)^-$	476.2410	6.67	0.072	31.429	0.035	223.68	0.25	457.2275	0.0003	1738	13	100	Y
M1T Glucuronide	$C_{26}H_{38}O_8$	$(M-H-H2O)^-$	478.2567	4.775	0.094	30.919	0.06	218.72	0.41	459.2398	0.0020	1066	14	100	Y
THMT-13 Glucuronide	$C_{26}H_{40}O_8$	$(M-H-H2O)^-$	480.2723	11.194	0.028	31.258	0.148	222.03	0.82	461.2546	0.0011	1114	14	100	Y
THMT Glucuronide	$C_{26}H_{42}O_8$	$(M-H-H2O)^-$	482.2880	11.170	0.020	29.236	0.152	207.19	0.54	463.2699	0.0012	988	11	91.7	Y
THMT Glucuronide	$C_{26}H_{42}O_8$	$(M-H-H2O)^-$	482.2880	11.203	0.027	30.823	0.155	218.45	1.00	463.2701	0.0010	4826	33	100	Y
THMT bis-Sulfate	$C_{20}H_{34}O_8S_2$	$(M-H)^-$	466.1695	5.731	0.109	28.054	0.073	198.62	0.43	465.1576	0.0011	2064	25	100	N
M1T Glucuronide	$C_{26}H_{38}O_8$	$(M-H)^-$	478.2567	10.836	0.056	31.685	0.16	224.62	0.4	477.2496	0.0010	570	7	100	Y
M1T Glucuronide	$C_{26}H_{38}O_8$	$(M-H)^-$	478.2567	8.747	0.037	29.261	0.086	208.14	0.61	477.2516	0.0007	372	3	82.4	Y
M1T Glucuronide	$C_{26}H_{38}O_8$	$(M-H)^-$	478.2567	9.497	0.085	30.152	0.09	213.16	0.65	477.2518	0.0008	1523	5	90.4	Y
M1T Glucuronide	$C_{26}H_{38}O_8$	$(M-H)^-$	478.2567	8.371	0.077	30.485	0.058	216.61	0.23	477.2527	0.0013	1813	8	99.5	Y
THMT-13 Glucuronide	$C_{26}H_{40}O_8$	$(M-H)^-$	480.2723	10.713	0.071	29.188	0.145	206.82	0.42	479.2647	0.0008	8166	28	100	Y
THMT-13 Glucuronide	$C_{26}H_{40}O_8$	$(M-H)^-$	480.2723	5.436	0.067	30.512	0.017	216.93	0.12	479.2649	0.0006	396	5	96.3	Y
THMT-13 Glucuronide	$C_{26}H_{40}O_8$	$(M-H)^-$	480.2723	9.576	0.102	30.297	0.146	214.08	1.07	479.2653	0.0015	749	7	84.9	Y
THMT Glucuronide	$C_{26}H_{42}O_8$	$(M-H)^-$	482.2880	6.357	0.098	29.834	0.016	210.83	0.08	481.2789	0.0003	1082	9	98.7	Y
THMT Glucuronide	$C_{26}H_{42}O_8$	$(M-H)^-$	482.2880	9.959	0.073	30.281	0.191	214.67	1.61	481.2805	0.0013	1505	19	92.9	Y
THMT Glucuronide	$C_{26}H_{42}O_8$	$(M-H)^-$	482.2880	10.486	0.171	30.717	0.176	217.25	1.27	481.2805	0.0012	2608	17	99.1	Y
THMT Glucuronide	$C_{26}H_{42}O_8$	$(M-H)^-$	482.2880	10.953	0.054	30.247	0.151	214.38	0.39	481.2806	0.0013	287	4	81.8	Y
THMT Glucuronide	$C_{26}H_{42}O_8$	$(M-H)^-$	482.2880	6.771	0.026	29.630	0.030	210.69	0.21	481.2809	0.0003	389	5	84	Y

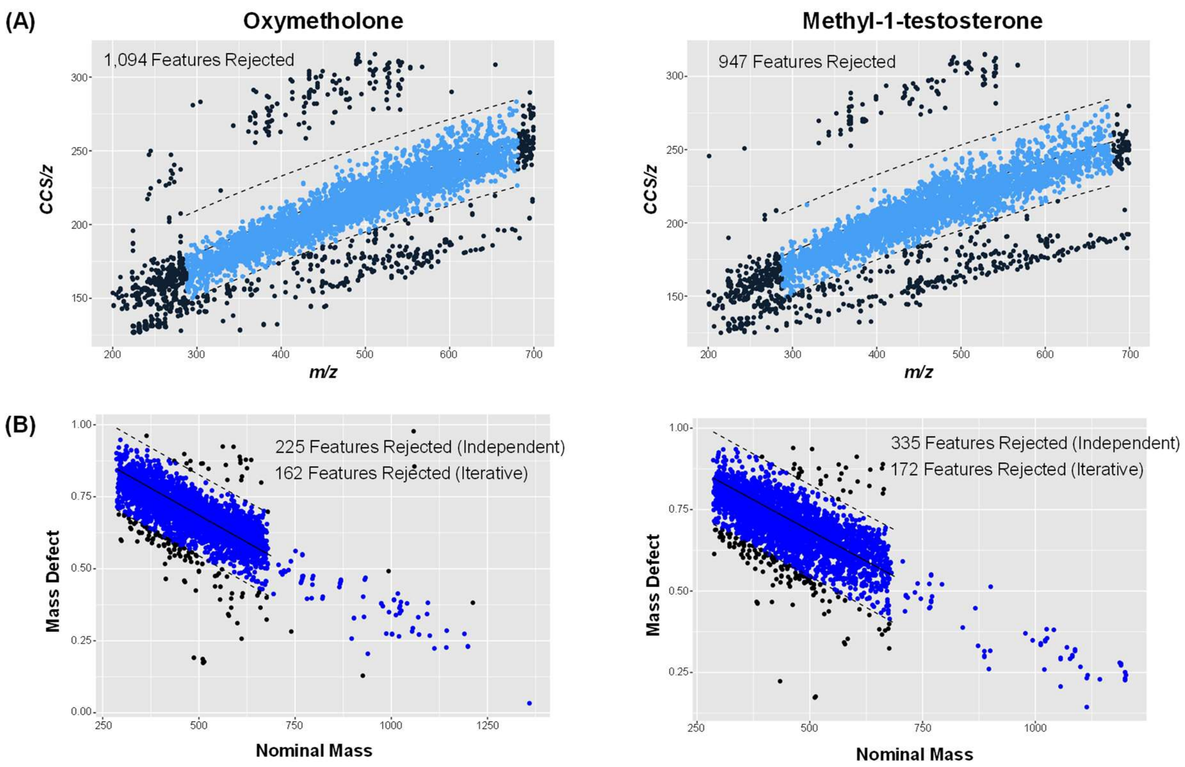

Figure S1. Overview of data analysis for the fully untargeted approach showing (**A**) conformational space plots (*m/z* vs CCS) and (**B**) mass defect plots for oxymetholone (left) and methyl-1-testosterone (right). Predictive intervals (99%) are illustrated by the dashed lines above and below the mean (solid line) based upon steroid entries in the Unified CCS Compendium.

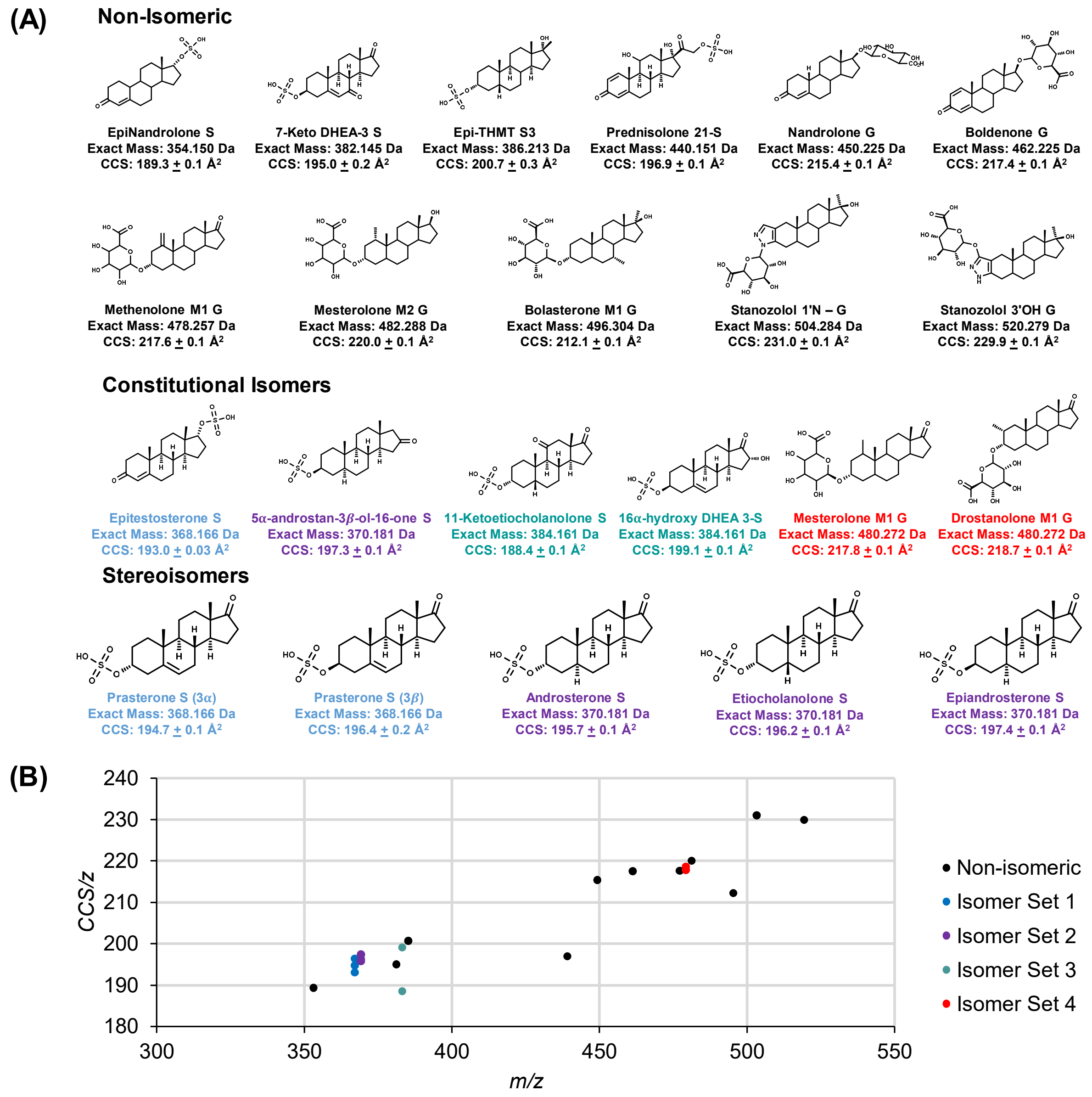

Figure S2. (A) Structures for phase II anabolic-androgenic steroid standards. Structures are grouped by non-isomeric, constitutional isomer, and stereoisomer constituents. Isomers are color-coded by the text. S and G abbreviations denote a sulfate or glucuronide phase II conjugate, respectively. Exact mass and CCS values of each standard are annotated below each structure. **(B)** Conformational space plot showing where isomer sets occur in *CCS/z* vs *m/z* space.

(A)

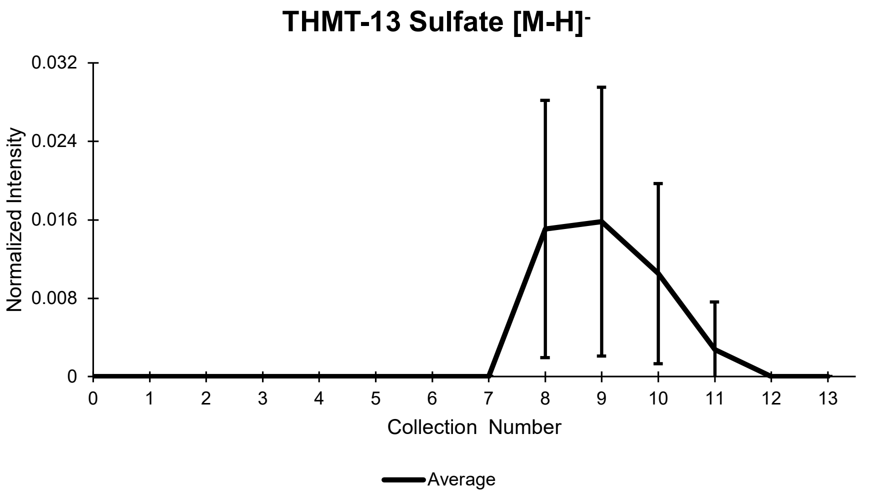

(B)

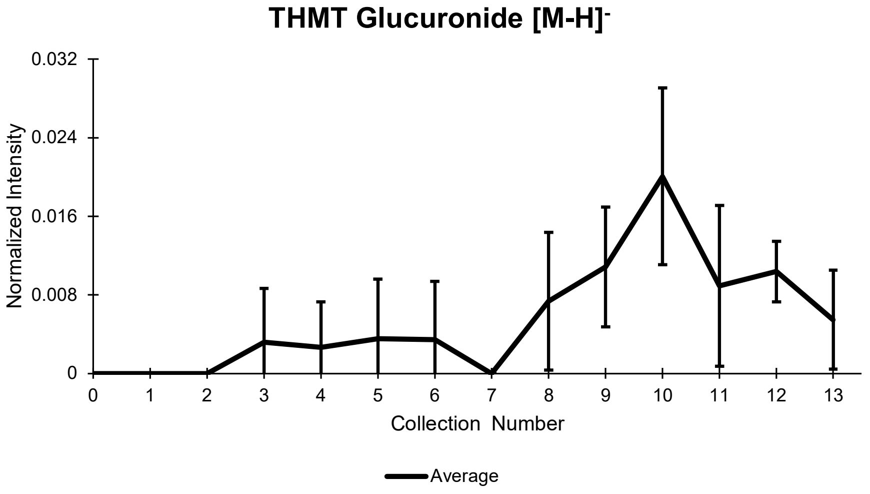

Figure S3. Temporal response profiles for (**A**) sulfate and (**B**) glucuronide metabolites of methyl-1-testosterone (M1T). Standard deviation is calculated from technical replicates (three instrument injections). Intensity values are normalized to the total ion chromatogram.

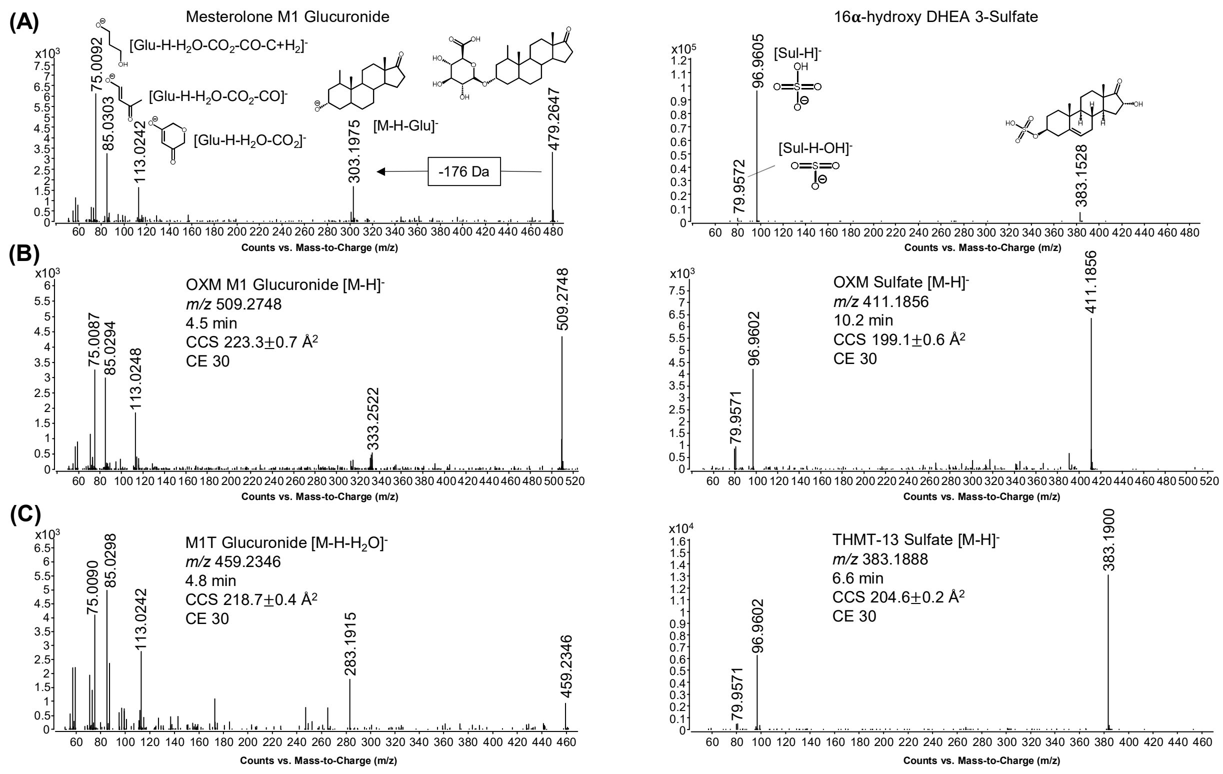

Figure S4. LC-IM-MS/MS spectra for two example AAS standards, Mesterolone M1 Glucuronide and 16α-hydroxy DHEA 3-Sulfate, spiked in blank urine (**A**) and putatively identified phase II metabolites of oxymetholone (**B**) and methyl-1-testosterone (**C**) indicating the presence of glucuronide- and sulfate-containing compounds. Structures for the known standards are denoted above each respective peak . Feature summary information used for the inclusion list is annotated for putatively identified OXM and M1T metabolites.
